# A Systematic Review of Self-Care Interventions for African American Family Caregivers

**DOI:** 10.1093/geroni/igab046.1369

**Published:** 2021-12-17

**Authors:** Kathy Wright, Eunjung Ko, Karen Moss, Tara O'Brien, Loren Wold, Karen Rose

**Affiliations:** 1 The Ohio State University, Columbus, Ohio, United States; 2 The Ohio State University, Center for Healthy Aging, Self-Management, and Complex Care College of Nursing, Columbus, Ohio, United States; 3 The Ohio State Univesity, College of Nursing andDepartment of Physiology and Cell Biology, Columbus, Ohio, United States

## Abstract

African Americans (AA) family caregivers provide the bulk of unpaid care to persons living with dementia (PLWD). This role leaves little time for the adoption of self-care behaviors—critical to the prevention and management of chronic diseases. In this systematic review, we appraise caregiver self-care interventions that include AA caregivers of PLWD. PubMed, CINAHL, PsycINFO, Cochrane, and Embase databases were searched. Terms included AA/Black, self-care, caregiving, lifestyle, intervention, psychological stress, and faith/community. We initially found 250 references and after application of exclusion criteria and removal of redundant references, 18 articles were analyzed. Most interventions focused on impacting levels of caregiver burden, depression, physical activity, anxiety, or wellbeing. Psychoeducation, physical activity, and spiritually-focused interventions were most effective in improving outcomes in caregivers. Future studies should examine the impact of interventions on the prevention and management of chronic disease in AA caregivers of PLWD.

